# Chest compression synchronized ventilation versus chest compression superimposed by sustained inflation in asphyxiated newborn piglets: a randomized animal trial

**DOI:** 10.1186/s40635-026-00920-6

**Published:** 2026-06-04

**Authors:** Raza Hyderi, Shrieya Praveen, Megan O’Reilly, Marwa Ramsie, Tze-Fun Lee, Georg M. Schmölzer

**Affiliations:** 1 Centre for the Studies of Asphyxia and Resuscitation, Neonatal Research Unit, Royal Alexandra Hospital, 10240 Kingsway Avenue NW, Edmonton, AB T5H 3V9 Canada; 2 Department of Pediatrics, University of Alberta, Edmonton, AB Canada

**Keywords:** Newborn, Chest compression, Asphyxia, Delivery room

## Abstract

**Background:**

Guidelines on neonatal resuscitation recommend 90 chest compressions (CCs) and 30 ventilations (3:1 C:V) per minute in newborns. We have described an alternative resuscitation strategy where CCs are superimposed with sustained inflation (CC + SI), which allows for passive ventilation during compression. A more recent strategy is CCs with synchronized ventilation (CCSV), in which a ventilator flow sensor recognizes airflow during the downward phase of compression and thereby triggers an inflation. No study has compared CCSV with CC + SI in an asphyxiated newborn piglet model. Newborn piglets (*n* = 8/group) were anesthetized, intubated, instrumented, and exposed to 45 min of normocapnic hypoxia, followed by asphyxia and asystolic cardiac arrest. Piglets were randomized to CCSV or CC + SI. Hemodynamic and respiratory parameters were continuously measured.

**Results:**

Sixteen neonatal mixed-breed piglets (1–3 days of age, weighing 1.7–2.8 kg) were randomized to CCSV or CC + SI. Median (IQR) time to ROSC was 68 (50–125) s with CCSV and 71 (60–178) s with CC + SI (*p* = 0.537). The rate of ROSC with CCSV compared to CC + SI was 6/8 (75%) vs. 5/8 (63%), respectively, *p* = 1.000. CCSV had significantly higher peak inflation pressure (45 vs. 36 cmH_2_O) and lower positive end-expiratory pressure (5.3 vs. 37 cmH_2_O) compared to CC + SI (both *p* < 0.001); tidal volumes were not significantly different.

**Conclusions:**

Use of CCSV did not result in a faster time to ROSC compared to CC + SI, and survival rates and physiological stability did not differ significantly.

## Introduction

Approximately 0.1–1% of term infants require cardiopulmonary resuscitation (CPR) at birth primarily due to asphyxia, which necessitates a resuscitation approach that prioritizes ventilation [1, 2]. The current consensus on science and treatment recommendations recommends a 3:1 compression to ventilation ratio (3:1 C:V), which consists of 90 chest compressions (CCs) and 30 ventilations per minute [1, 2]. However, the most effective CPR approach for newborn infants remains unknown.

Previous neonatal animal studies compared different C:V ratios, including 2:1, 4:1, 9:3, and 15:2, and reported no difference in survival or time to return of spontaneous circulation (ROSC) [3–5]. While all studies provided high-quality CCs, an overlooked effect is lung de-recruitment, which occurs with every compression [6, 7]. Indeed, during the downward phase of compression, air is forced out of the lung, thereby resulting in lung de-recruitment with reduced alveolar surface for gas exchange [7]. This could result in longer duration of CCs, which is associated with increased risk of mortality and long-term neurological impairment [8–11].

Therefore, approaches that combine high-quality CCs with improved ventilation strategies to prevent lung de-recruitment might improve outcomes during neonatal CPR. We have previously reported that chest compression superimposed with sustained inflation (CC + SI) delivers a constant high distending pressure during continuous CCs [12–17]. During CC + SI, the high distending pressure allows air to passively flow back into the lung during chest recoil with each compression. Our animal and human studies have reported a significantly faster time to ROSC, reduced mortality, higher intrathoracic pressure, and improved hemodynamic parameters using CC + SI [12–17]. A more recent alternative might be chest compression synchronized ventilation (CCSV) [18–22], a novel resuscitation technique that may also overcome lung de-recruitment. CCSV can be activated in the MEDUMAT Standard^2^ Ventilator (Weinmann Emergency Medical Technology, Hamburg, Germany) by pressing a button. In the CCSV mode, the flow sensor detects compression-induced expiratory flow (air forced out of the lungs) and the associated rise in airway pressure during the compression. Within ~ 200–345 ms of the compression, the ventilator initiates an inflation during the down stroke of each compression, with exhalation occurring during chest recoil. If CC flow is not detected by the ventilator, the device switches to volume-controlled intermittent positive-pressure ventilation as a back-up mode while alerting the user. This CCSV technique may maintain lung recruitment, improve gas exchange, and oxygenation. Previous studies using CCSV in adult piglets reported conflicting results, with one study reporting improved partial pressure of arterial oxygen (PaO_2_) and lower partial pressure of arterial carbon dioxide (PaCO_2_), while another study reported no difference in either parameter [18–20]. As ventilation is the cornerstone of neonatal resuscitation, CCSV might further improve outcomes compared to CC + SI. We aimed to compare CCSV with CC + SI in a piglet model of neonatal asphyxia. We hypothesized that CCSV, compared to CC + SI, would reduce the time to ROSC in asphyxiated neonatal piglets.

## Methods

**Fig. 1:**
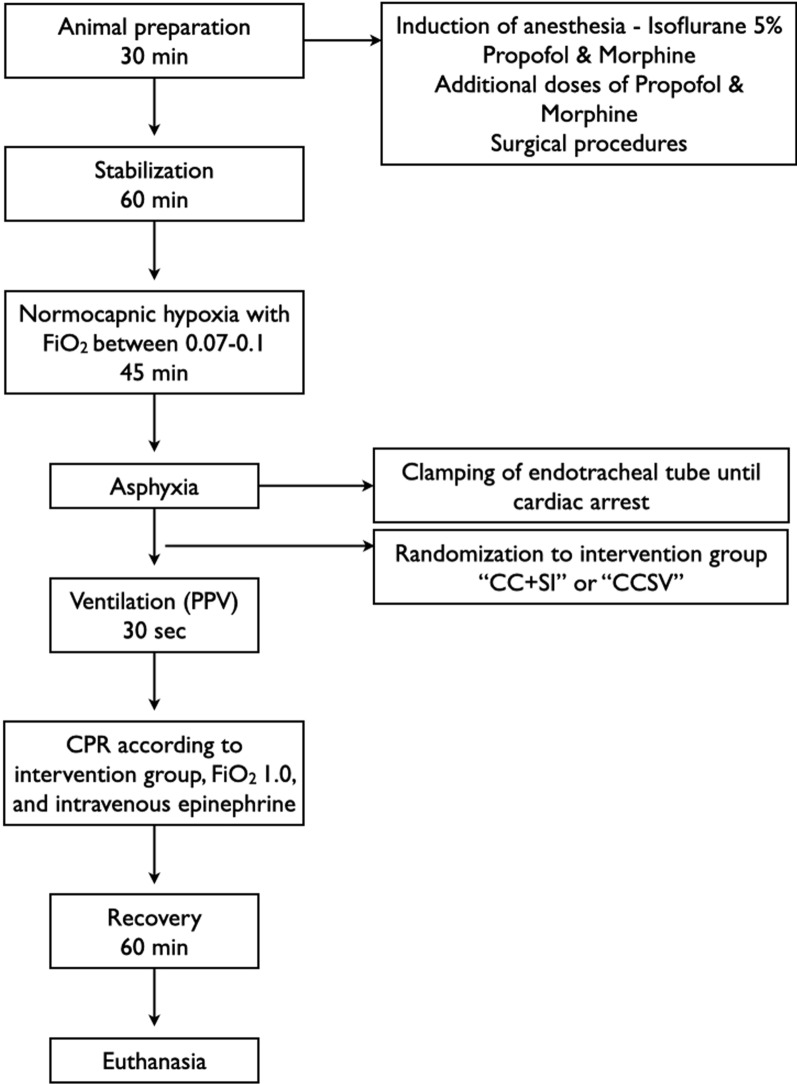
Study flowchart. *PPV* positive-pressure ventilation, *CPR* cardiopulmonary resuscitation, *CCSV* chest compression synchronized ventilation, *CCSI* chest compression superimposed by sustained inflation, *FiO*_*2*_ fraction of inspired oxygen

All experiments were conducted in accordance with the guidelines and approval of the Animal Care and Use Committee (Health Sciences), University of Alberta [AUP00002920], presented according to the ARRIVE 2.0 guidelines [23], and registered at preclinicaltrials.eu (PCTE0000637). A graphical display of the study protocol is presented in Fig. 1.

### Inclusion and exclusion criteria

Neonatal mixed-breed piglets (1–3 days of age, weighing 1.7–2.8 kg) obtained on the day of experimentation from the University Swine Research Technology Centre were included. There were no exclusion criteria.

### Randomization

Piglets were randomly allocated to either CCSV or CC + SI. Allocation was block randomized with variable-sized blocks using a 1:1 allocation using a computer-generated randomization program (https://www.randomizer.org). Sequentially numbered, sealed, brown envelopes containing the allocation were opened during the experiment.

### Blinding

Owing to the nature of the intervention, it was not feasible to blind the team to the allocated intervention. However, the randomization process enabled the concealment of the intervention until cardiac arrest was confirmed (by the co-author GMS). The statistical analysis was blinded to group allocation and only unblinded after the statistical analysis was complete.

### Sample size and power estimates

Our primary outcome measure was time to ROSC. Our previous studies showed the mean (SD) time to ROSC with CC + SI was 256 (37). We hypothesized that a sample size of 16 piglets (8 per group) would be sufficient to detect a 20% decrease in time to ROSC with 80% power and a 2-tailed alpha error of 0.05.

### Animal preparation

Piglets were instrumented as previously described with modifications [12, 24], as detailed in brief below. Following induction of anesthesia using isoflurane, piglets underwent a tracheotomy for intubation. Pressure-controlled ventilation (Sechrist Infant Ventilator Model IV-100; Sechrist Industries, Anaheim, CA) was commenced at a respiratory rate of 16–20 breaths/min and a pressure of 20/5 cmH_2_O. Oxygen saturation was maintained within 90–100%, while glucose was provided via an intravenous infusion of 5% dextrose at 10 mL/kg/h. Intravenous propofol (5–10 mg/kg/h) and morphine (0.1 mg/kg/h) were provided during the experiment to maintain anesthesia, and additional intravenous doses of propofol (1–2 mg/kg) and morphine (0.05–0.1 mg/kg) were administered as needed. Piglets’ normothermic body temperature of 38.5–39.5 °C was maintained with a heating pad and overhead warmer.

### Hemodynamic parameters

5-French Argyle^®^ (Klein-Baker Medical Inc., San Antonio, TX) triple-lumen and single-lumen catheters were inserted via the right femoral vein and artery, respectively. The femoral venous catheter was used for administration of fluids and medications and to continuously measure central venous pressure. The arterial catheter was used for continuous arterial blood pressure monitoring in addition to arterial blood gas measurements. A real-time ultrasonic flow probe (2 mm; Transonic Systems Inc., Ithaca, NY) encircled the right common carotid artery to measure cerebral blood flow. A 3-lead ECG (Hewlett Packard 78833B monitor, Hewlett Packard Co., Palo Alto, California) using adhesive leads was placed on the skin at the right forelimb, left forelimb, and left hind limb.

Following surgical instrumentation, piglets were placed in the supine position and allowed to recover from surgical instrumentation until baseline hemodynamic measures were stable (minimum one hour). Ventilator rate was adjusted to maintain the partial arterial CO_2_ between 35 and 45 mmHg, as determined by periodic arterial blood gas analysis. A Hewlett Packard 78833B monitor (Hewlett Packard Co., Palo Alto, CA) was used for the continuous measurement of heart rate, mean systemic arterial pressure, systemic systolic and diastolic arterial pressure, and percutaneous oxygen saturation throughout the experiment.

### Respiratory function monitor

A respiratory function monitor (NM3, Respironics, Philips, Andover, MA) was used to continuously measure V_T_, airway pressures, and gas flow. The NM3 flow sensor has a fixed-orifice pneumotach, which uses the pressure difference to calculate the gas flow passing through the sensor, which is then translated into the inspiratory and expiratory V_T_. The sensor was placed between the endotracheal tube and the ventilation device. V_T_ was calculated by integrating the flow signal [25]. The accuracy for gas flow is ± 0.125 L/min [26].

### Cerebral perfusion

Cerebral oxygenation (crSO_2_) was measured using the Invos^™^ Cerebral/Somatic Oximeter Monitor (Invos 5100, Somanetics Corp., Troy, MI), which calculates crSO_2_ and expresses values as the percentage of oxygenated hemoglobin (oxygenated hemoglobin/total hemoglobin) [27]. The sensor was placed on the right forehead of the piglet and secured with wrap and tape. A slim cap provided light shielding. Regional oxygen saturation values were recorded every second at a sample rate of 0.13 Hz.

### Experimental protocol

Following surgical instrumentation and stabilization, piglets were exposed to 45 min of normocapnic hypoxia, which was followed by asphyxia. Asphyxia was achieved by disconnecting the ventilator and clamping the endotracheal tube until cardiac arrest. Cardiac arrest was defined as zero carotid blood flow and no audible heartbeat during auscultation. A numbered, sealed brown envelope containing the allocation “CCSV” or “CC + SI” was opened.

Thirty seconds after confirmation of cardiac arrest, positive-pressure ventilation (PPV) was provided for 30 s. If randomized to CC + SI, PPV was provided with a Neopuff T-Piece (Fisher & Paykel, Auckland, New Zealand) with 100% oxygen, peak inspiratory pressure of 30 cmH_2_O, positive end-expiratory pressure of 5 cmH_2_O, and gas flow of 10 L/min. If randomized to CCSV, PPV was provided with the MEDUMAT Standard^2^ with 100% oxygen, peak inflation pressure of 40 cmH_2_O, positive end-expiratory pressure of 5 cmH_2_O.

After 30 s of PPV, CCs were started and were performed using the two-thumb CC technique at a rate of 120 compressions/min (guided by a metronome).

If randomized to CC + SI, SIs during CC were delivered using a T-Piece with a peak inflating pressure of 30 cmH_2_O, a positive end-expiratory pressure of 5 cmH_2_O, and 100% oxygen for 30 s. The SI was interrupted for 1 s before a further 30 s of SI was provided.

If randomized to CCSV, the CCSV button on the MEDUMAT Standard^2^ ventilator was pressed to activate CCSV mode. In the CCSV mode, the ventilator synchronizes inflations with the down stroke of each chest compression. Synchronization is achieved when the ventilator’s flow sensors detect compression-induced expiratory flow (air forced out of the lungs) and the associated rise in airway pressure during the compression. The ventilator then delivers a positive-pressure inflation targeting a pre-set peak inflation pressure of 40 or 60 cmH_2_O, initiated within ~200–345 ms of the compression. During chest recoil, exhalation occurs. Positive end-expiratory pressure is adjustable. A dedicated flow-trigger sensitivity control, used solely to detect compression-induced airflow, is adjustable to optimize synchronization and does not otherwise alter ventilator behavior. The trigger sensitivity has five levels, with level 1 being the most sensitive and level 5 the least sensitive. CCSV mode commenced at trigger level 2, and the trigger level was manually adjusted between 1 and 5 to ensure optimized synchronization between ventilation and CCs. The CCSV mode is designed for patients ≥10 kg. During the study, CCSV was delivered with a peak inflating pressure of 40 cmH_2_O, a positive end-expiratory pressure of 5 cmH_2_O, and 100% oxygen.

CC + SI and CCSV were continued until ROSC or a maximum resuscitation time of 10 min, whichever occurred first. The cardio-resuscitative drug, epinephrine, was administered (0.02 mg/kg per dose) intravenously 2 min after the start of CCs and thereafter every 3 min until ROSC, with a maximum of three doses. Each dose of epinephrine was followed by a 3 mL normal saline flush. ROSC was defined as an unassisted heart rate > 100/min for at least 15 s, detected by ECG. After ROSC, piglets recovered and were monitored for 60 min while continuously ventilated. Blood gases were collected at defined cardiac arrest, immediately after ROSC, and after 60 min of recovery. At the end of experimentation, piglets were euthanized with an intravenous overdose of sodium pentobarbital (120 mg/kg).

### Tissue preparation and analysis

Tissue samples were only collected from piglets that survived one hour after ROSC. Following euthanasia, right lung tissue was collected. Lung tissue samples were snap-frozen in liquid nitrogen and stored at −80 °C. Tissue samples were homogenized in lysis buffer (0.5% Tween-20/PBS containing protease inhibitor cocktail), centrifuged (3,000xg for 10 min at 4 °C). The supernatants were collected. Protein concentration was quantified using the Bradford method. Evidence of lung injury was determined by quantification of the concentrations of the pro-inflammatory cytokines interleukin (IL)-6, IL-8, and tumor necrosis factor (TNF)-α in tissue homogenates by using commercially available ELISA kits as per manufacturer’s instructions (P6000B, P8000, PTA00; R&D Systems, Minneapolis, MN).

### Data collection and analysis

Demographics of study piglets were recorded. Transonic flow probe, heart rate, and pressure transducer outputs were digitized and recorded with LabChart^®^ programming software (ADInstruments, Houston, TX). Airway pressures, gas flow, tidal volume, and end-tidal CO_2_ were measured and analyzed using Flow Tool Physiologic Waveform Viewer (Philips Healthcare, Wallingford, CT). The data were tested for normality (Shapiro–Wilk and Kolmogorov–Smirnov tests) and compared using Student’s *t*-test for parametric, Mann–Whitney U-test for nonparametric comparisons of continuous variables, and Fisher exact test for categorical variables. The data are presented as mean (standard deviation, SD) for normally distributed continuous variables and median (interquartile range, IQR) when the distribution is skewed. *P*-values are 2-sided, and *p* < 0.05 was considered statistically significant. Statistical analyses were performed with SigmaPlot (Systat Software Inc., San Jose, CA).

## Results

Sixteen neonatal mixed-breed piglets were obtained on the day of the experiment (1–3 days of age, weighing 1.7–2.8 kg) and were randomly assigned to CCSV or CC + SI. Baseline characteristics are presented in Table 1 and blood gases at commencement of resuscitation (cardiac arrest), immediately after resuscitation, and 60 min after ROSC are presented in Table 2.

**Table 1:** Baseline characteristics

	CCSV (*n* = 8)	CC + SI (*n* = 8)	*p*-value
Age (days)	2.5 (1.3–3.0)	2.0 (2.0–2.8)	0.776
Weight (kg)	1.9 (1.8–2.2)	2.0 (1.7–2.1)	0.904
Gender (male/female)	6/2	8/0	0.467
Heart rate (bpm)	160 (148–232)	163 (153–205)	0.878
MAP (mmHg)	72 (67–76)	68 (63–86)	0.844
Carotid flow (mL/min)	41 (33–55)	40 (35–52)	0.878
Cerebral oxygenation (%)	50 (43–52)	45 (42–47)	0.217
pH	7.49 (7.42–7.58)	7.47 (7.44–7.50)	0.721
paCO_2_ (mmHg)	32 (29–35)	33 (30–37)	0.505
paO_2_ (mmHg)	73 (63–85)	65 (62–70)	0.234
Base excess (mmol/L)	1.4 (−2.2 to 4.8)	0.1 (−2.2 to 3.3)	0.798
Lactate (mmol/L)	5.7 (4.6–6.0)	5.8 (3.9–7.7)	0.764

Data are presented as median (IQR); *MAP* mean arterial blood pressure, *PaCO*_*2*_ partial pressure of arterial carbon dioxide, *PaO*_*2*_ partial pressure of arterial oxygen, *CCSV* chest compression synchronized ventilation, *CC* + *SI* chest compression superimposed by sustained inflation

**Table 2:** Blood gas changes throughout the experiment

	CCSV (*n* = 8)	CC + SI (*n* = 8)	*p*-value
Commencement of resuscitation			
Arterial pH	6.66 (6.61–6.85)	6.71 (6.69–6.78)	0.574
PaCO_2_ (torr)	82 (73–95)	71 (68–80)	0.105
PaO_2_ (torr)	5 (5–8)	5 (5–7)	0.959
Base excess (mmol/L)	−26 (−29 to −22)	−27 (−28 to −24)	0.878
Lactate (mmol/L)	20 (20–20)	20 (20–20)	1.000
Immediately after return of spontaneous circulation			
Arterial pH	6.86 (6.79–6.99)	6.78 (6.74–6.86)	0.151
PaCO_2_ (torr)	20 (18–33)	30 (22–35)	0.247
PaO_2_ (torr)	377 (250–479)	405 (331–443)	0.733
Base excess (mmol/L)	−28 (−30 to −26)	−30 (−30 to −28)	0.285
Lactate (mmol/L)	20 (20–20)	20 (20–20)	1.000
60 min after return of spontaneous circulation			
Arterial pH	7.17 (7.06–7.25)	7.00 (6.86–7.15)	0.141
PaCO_2_ (torr)	36 (35–37)	40 (31–51)	0.686
PaO_2_ (torr)	102 (82–140)	90 (71–99)	0.264
Base excess (mmol/L)	−15 (−20 to −11)	−21 (−25 to −18)	0.121
Lactate (mmol/L)	15 (13–17)	19 (16–20)	0.114

Data are presented as median (IQR); *PaCO*_*2*_ partial pressure of arterial carbon dioxide, *PaO*_*2*_ partial pressure of arterial oxygen, *CCSV* chest compression synchronized ventilation, *CC* + *SI* chest compression superimposed by sustained inflation

### Resuscitation

Median (IQR) time of asphyxia from endotracheal tube occlusion was 326 (275–405) and 281 (192–351) s with CCSV and CC + SI, respectively, *p* = 0.382 (Table 3). Median time (IQR) to ROSC among survivors was not significantly faster with CCSV compared to CC + SI (68 (50–125) vs. 71 (60–178) s, *p* = 0.537, Table 3). The rate of ROSC with CCSV compared to CC + SI was 6/8 (75%) vs. 5/8 (63%), respectively, *p* = 1.000 (Table 3). The median (IQR) duration of resuscitation, which includes piglets that did not achieve ROSC, with CCSV compared to CC + SI was 97 (54–486) vs. 178 (70–600) s, *p* = 0.442, respectively (Table 3).

**Table 3:** Characteristics of asphyxia, resuscitation, and survival of asphyxiated piglets

	CCSV (*n* = 8)	CC + SI (*n* = 8)	*p*-value
Asphyxia time (sec)	326 (275–405)	281 (192–351)	0.382
Heart rate (bpm)	78 (55–86)	77 (73–90)	0.677
Mean arterial pressure (mmHg)	20 (17–20)	20 (16–20)	0.657
Epinephrine doses (*n*)	0.5 (0–2.5)	1 (0–3)	0.645
Achieving ROSC ^†^	6 (75%)	5 (63%)	1.000
ROSC time (s)	68 (50–125)	71 (60–178)	0.537
CPR time (s)	97 (54–486)	178 (70–600)	0.442

Data are presented as median (IQR), unless indicated ^†^*n* (%); *CCSV* chest compression synchronized ventilation, *CC* + *SI* chest compression superimposed by sustained inflation, *CPR* cardiopulmonary resuscitation, *ROSC* return of spontaneous circulation

### Changes in hemodynamic parameters

**Fig. 2:**
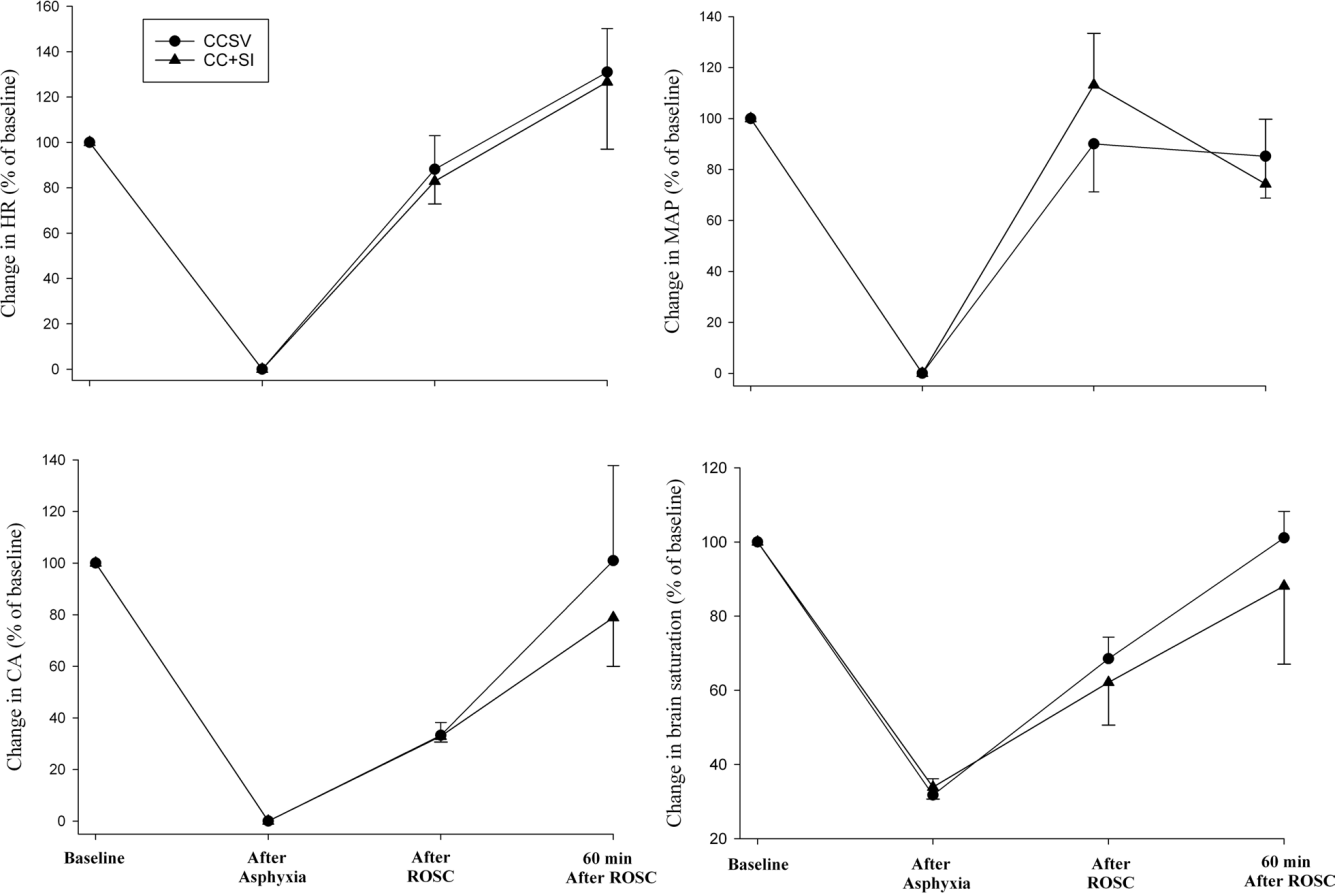
Hemodynamic parameters. Changes in heart rate, mean arterial pressure, carotid blood flow, and brain oxygen saturation throughout the experiment. Data are expressed as % change from baseline and presented as mean (SD). There were no statistically significant differences in hemodynamic parameters between CCSV and CC + SI groups. There were no statistically significant differences in hemodynamic parameters between CCSV and CC + SI groups. *CCSV* chest compression synchronized ventilation, *CC* + *SI* chest compression superimposed by sustained inflation, *HR* heart rate, *MAP* mean arterial pressure, *CA* carotid artery, *ROSC* return of spontaneous circulation

**Fig. 3:**
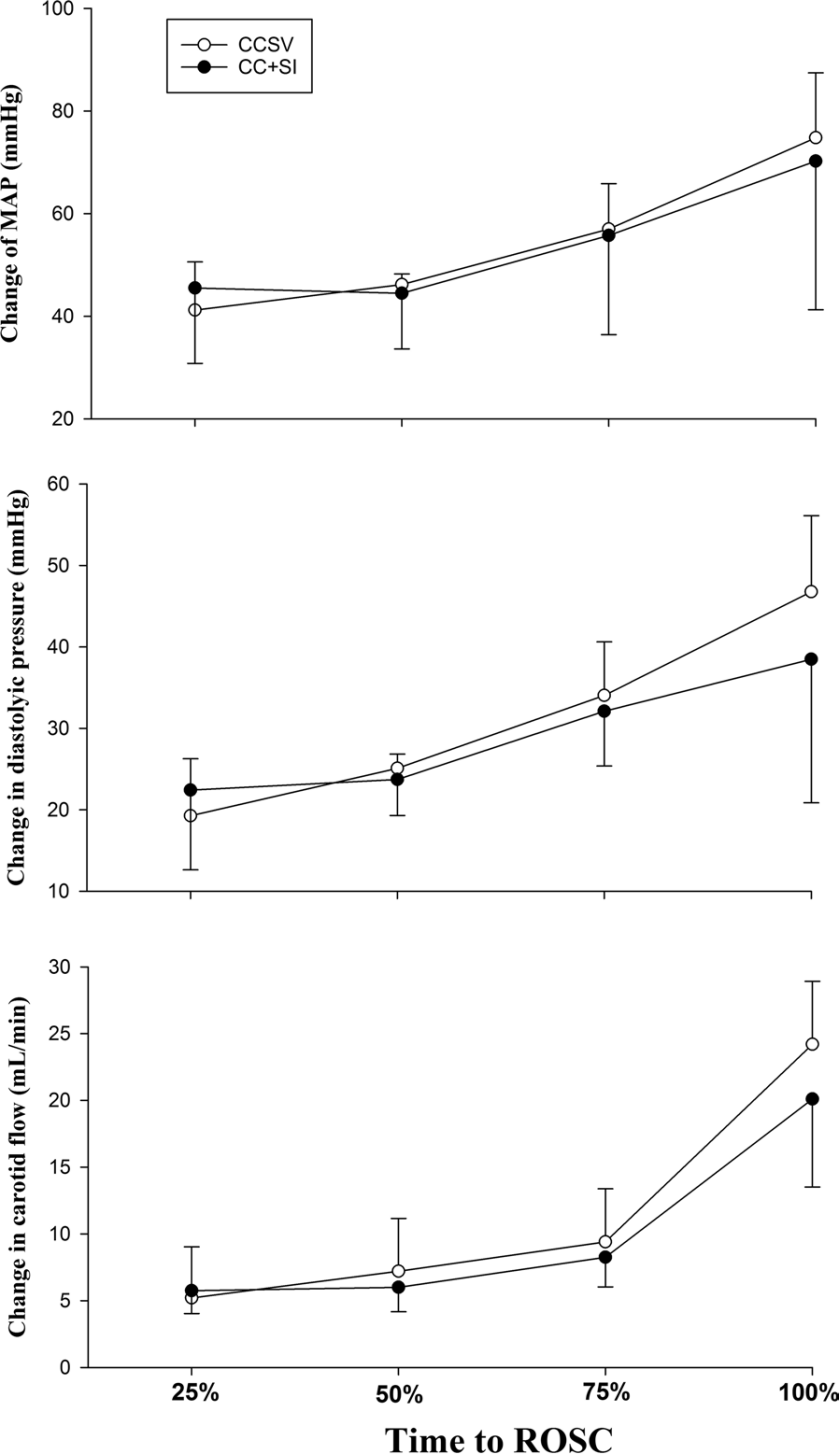
Hemodynamic changes during resuscitation following asystolic cardiac arrest. Changes in mean arterial pressure, diastolic blood pressure, and carotid blood flow. Data are expressed as % change from baseline and presented as mean (SD). Hemodynamic parameters are plotted against the proportion of resuscitation time and expressed as a percentage of the total resuscitation time. There were no statistically significant differences in hemodynamic parameters between CCSV and CC + SI groups. *CCSV* chest compression synchronized ventilation, *CC* + *SI* chest compression superimposed by sustained inflation, *MAP* mean arterial pressure, *ROSC* return of spontaneous circulation

Hemodynamic parameters, including heart rate, mean arterial pressure, carotid blood flow, and cerebral oxygenation, were not significantly different at baseline, after asphyxia, immediately after ROSC, and 60 min after ROSC between groups (Fig. 2). Hemodynamic changes in mean arterial pressure, diastolic pressure, and carotid blood flow during resuscitation in piglets that achieved ROSC are presented in Fig. 3. There were no statistically significant differences between CCSV and CC + SI groups.

### Respiratory parameters

**Fig. 4:**
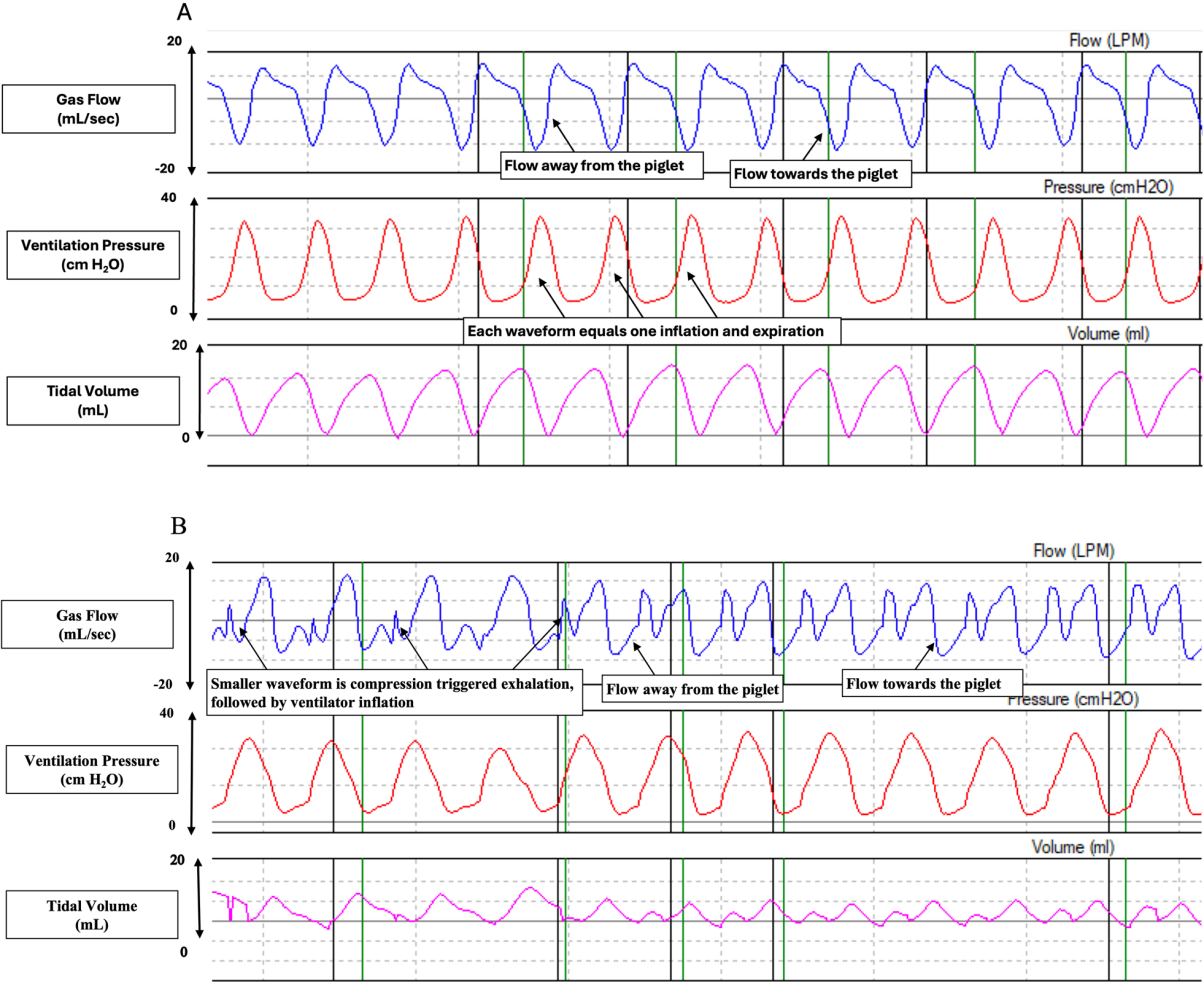
Respiratory waveforms during resuscitation. Respiratory waveforms during resuscitation in CC+SI (**A**) and CCSV (**B**) groups showing gas flow (mL/min), ventilation pressure (cmH2O), and tidal volume (ml)

Respiratory waveforms during resuscitation in the CCSV and CC + SI groups are presented in Fig. 4. Respiratory parameters are presented in Table 4. The mean (SD) peak inflation pressure was significantly higher (*p* = 0.0007) and the positive end-expiratory pressure was significantly lower with CCSV compared to CC + SI (*p* < 0.0001) (Table 4). There was no significant difference in tidal volume and minute ventilation with CCSV vs. CC + SI (both *p* = 0.073) (Table 4).

**Table 4:** Respiratory parameters

	CCSV (*n* = 8)	CC + SI (*n* = 8)	*p*-value
Ventilation rate (/min)	115 (3)	116 (4)	0.141
Peak inflation pressure (cmH_2_O)	44.8 (3.1)	36.4 (4.3)	0.0007
Positive end-expiratory pressure (cmH_2_O)	5.33 (0.23)	36.6 (4.6)	< 0.0001
End-tidal CO_2_ (mmHg)	13.8 (6.8)	9.6 (3.5)	0.157
Tidal volume (mL/kg)	6.35 (1.3)	5.28 (0.85)	0.073
Minute ventilation (mL/kg/min)	653 (225)	475 (113)	0.073

Data are presented as mean (SD); *CCSV* chest compression synchronized ventilation, *CC* + *SI* chest compression superimposed by sustained inflation

### Lung inflammation

**Fig. 5:**
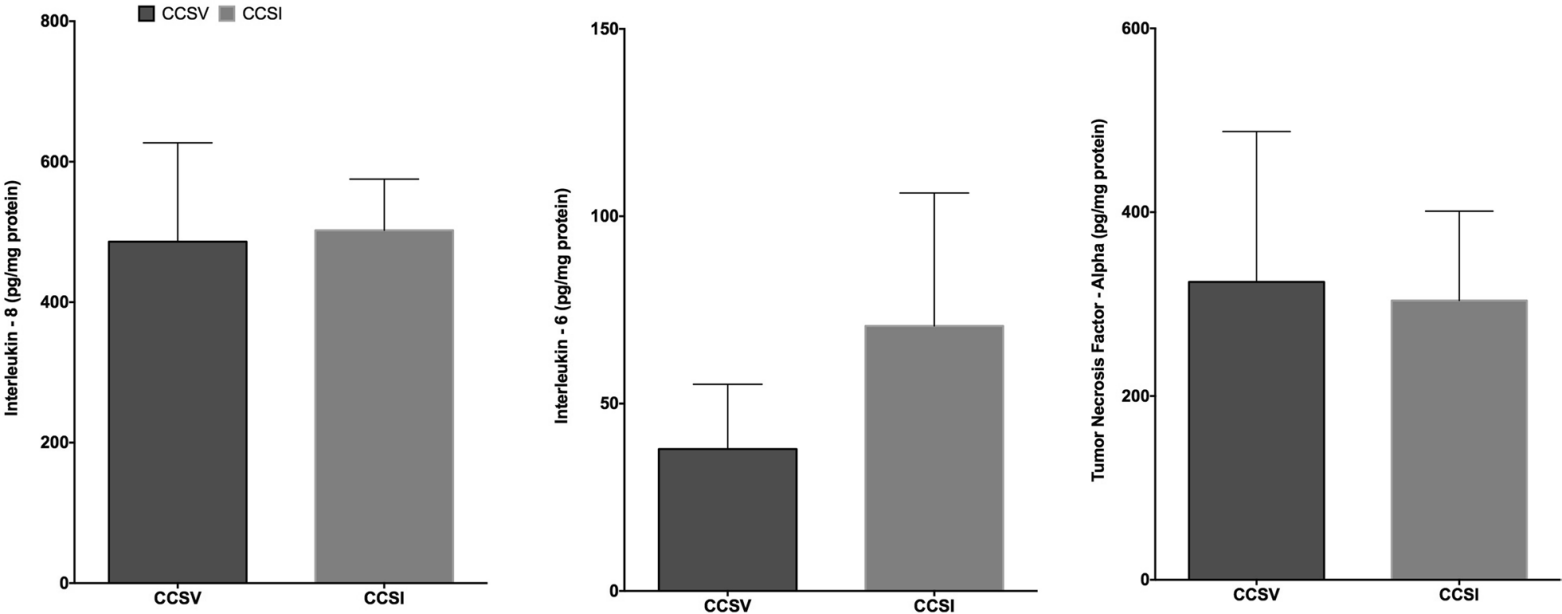
Interleukin (IL)-8, IL-6, and tumor necrosis factor-alpha. Concentrations of pro-inflammatory markers IL-8, IL-6, and TNF-alpha in lung tissue homogenates, expressed relative to lung protein concentration. *CCSV* chest compression synchronized ventilation, *CCSI* chest compression superimposed by sustained inflation

There were no significant differences in the expression of pro-inflammatory cytokines interleukin-6 and -8, and tumor necrosis factor-alpha in lung tissue homogenates between CCSV and CC + SI groups (Fig. 5).

## Discussion

Asphyxia, caused by impaired gas exchange with concurrent hypoxemia and hypercapnia, is the leading cause of neonatal cardiac arrest. Current consensus of science and treatment recommendations and resuscitation guidelines recommend administering neonatal CPR with the 3:1 C:V method [1, 2]. However, preclinical work has identified a net loss of lung volume when using 3:1 C:V, leading to possible lung de-recruitment [6]. Lung de-recruitment can compromise gas exchange and prolong time to ROSC; longer ROSC times are associated with higher mortality and worse long-term neurologic and health outcomes. Alternative ventilation strategies that prevent lung de-recruitment during neonatal CPR might improve outcomes. This study investigated two alternative CPR techniques, CCSV and CC + SI, in a neonatal piglet model of asystolic cardiac arrest. The results of our study can be summarized as follows: (1) CCSV was not superior to CC + SI in terms of time to ROSC, survival rate, and hemodynamic recovery; (2) the delivered tidal volume and minute volume were similar between techniques, despite a significant difference in delivered respiratory pressures; and (3) levels of pulmonary pro-inflammatory markers were comparable between CCSV and CC + SI.

Across five investigations in adult swine and a small clinical pilot, CCSV delivers stronger intraprocedural physiology but variable effects on ROSC [18–22]. In a 24-pig RCT, CCSV achieved higher PaO_2_, lower PaCO_2_, higher mean arterial pressure, and near-normal mixed-venous pH versus IPPV or bilevel, yet ROSC rates were comparable (IPPV 5/8; bilevel 6/8; CCSV 4/7) [19]. A prolonged-arrest model comparing CCSV with continuous compressions plus asynchronous ventilation showed no significant differences in gas exchange or hemodynamics [20]. A crossover porcine study confirmed that multiple CCSV presets increased PaO_2_ and prevented the arterial-pressure decline seen with IPPV [18]. Notably, combining CCSV with aortic balloon occlusion enhanced perfusion, produced ROSC in 7/7 animals, and lessened post-resuscitation organ injury versus IPPV with aortic balloon occlusion [22]. In contrast, a randomized out-of-hospital cardiac arrest pilot reported significantly improved oxygenation and ventilation in patients undergoing CPR with CCSV; however, there was no between-group superiority over IPPV [21]. Consistent with these data, we observed no difference in time to ROSC or mortality, and in blood gas, ventilation or hemodynamic parameters.

High-quality chest compressions given hard and fast, and with minimal interruptions are central to achieving ROSC. A less appreciated downside, however, is pulmonary de-recruitment: each compression forces air out of the lungs (a forced exhalation), promoting atelectasis [6, 7, 28]. In adult pigs undergoing 7 min of compression-only CPR, CT scans showed lung atelectasis rising from 25% pre-CPR to 75% post-CPR [28], and in neonatal piglets we observed a mean tidal-volume loss of 4.5 mL/kg with each 3:1 C:V cycle [6]. Adult swine and clinical studies report promising gains in ventilation and hemodynamics with CCSV [18, 19, 21, 22], though one recent animal study noted more pneumothoraces than controls and prior CCSV work [20]. In our study, autopsy found no pneumothoraces, and lung-injury biomarkers were similar between CCSV and CC + SI, indicating no added lung injury. The similarity between CCSV and CC + SI in terms of pro-inflammatory cytokine expression in lung tissue after achieving ROSC in our study indicates that CCSV does not promote more acute lung injuries than CC + SI. Notably, Kopra et al. performed ~ 35 min of CPR [20], whereas our resuscitations were much shorter (median ~ 70 s).

CCSV delivers ventilator-triggered inflations on the compression down stroke with default peak inflation pressures of 40 or 60 cmH_2_O. In our study, we used a peak inflation pressure of 40 cmH_2_O for CCSV vs. 30 cmH_2_O with CC + SI; despite the higher peak inflation pressure, tidal volume delivery and lung-injury biomarkers were unchanged, suggesting no added barotrauma. Although current neonatal guidelines suggest peak inflation pressures of 20–30 cmH_2_O, observational data indicate 30–40 cmH_2_O may be needed for initial aeration [29, 30], and we previously showed a peak inflation pressure ≥ 25 cmH_2_O is required to achieve adequate tidal volume delivery with CC + SI [31]. Positive end-expiratory pressure was lower with CCSV, due to differences in both techniques, and this was also not correlated with lung injury.

Although time to ROSC did not differ, this likely reflects similar compression–ventilation mechanics rather than inadequate ventilation. CCSV delivers active inflations during the downstroke [18–22], whereas CC + SI achieves passive ventilation during recoil [12–17]; both produced comparable tidal volumes, minute ventilation, blood gases, and hemodynamic parameters. Both techniques align with the thoracic pump model, in which CPR-induced intrathoracic pressure drives forward flow [32–35]. We previously showed CC + SI raises intrathoracic pressure versus 3:1 C:V [36, 37], and Kill et al. reported higher mean arterial pressure and oxygenation with CCSV via synchronized pressure-augmenting inspirations [18, 19].

### Limitations

Our use of a piglet asphyxia model is a considerable strength of this translational study, because this model closely mimics delivery room events with a gradual onset of severe asphyxia leading to cardiac arrest. However, several limitations should be considered before general application of CCSV in future clinical neonatal resuscitation trials. Our model uses piglets that have undergone the fetus to neonate transition and do not possess fetal features (i.e., no lung fluid present or a patent ductus arteriosus). Additionally, our model uses piglets that were sedated/anesthetized and intubated with a tightly sealed endotracheal tube to prevent a leak, which may not occur in the delivery room. Furthermore, we did not directly measure intrathoracic pressure in this study. Cerebral oxygenation measurements with the Invos^™^ Cerebral/Somatic Oximeter Monitor may be influenced by extracerebral tissue owing to the size of the piglet brain. Nevertheless, our findings remain relevant despite these limitations, because several previous studies with our post-transitional model have been successfully translated into neonatal clinical randomized trials [13, 17, 38].

## Conclusions

In a neonatal porcine model of CPR, use of CCSV did not result in a faster time to ROSC compared to CC + SI. Furthermore, mortality, hemodynamic changes, and pulmonary inflammatory markers did not differ significantly between CCSV and CC + SI.

## Data Availability

The datasets generated and analyzed for this study are available from the corresponding author (GMS), upon reasonable request.
